# High-resolution X-ray diffraction and imaging

**DOI:** 10.1107/S0021889813016415

**Published:** 2013-07-18

**Authors:** Paul F. Fewster, Marina V. Baidakova, Reginald Kyutt

**Affiliations:** a PANalytical Research Centre, Sussex Innovation Centre, Science Park Square, Falmer, Brighton, East Sussex BN1 9SB, UK; b Ioffe Physical Technical Institute, Russian Academy of Sciences, St Petersburg, Russian Federation

**Keywords:** high-resolution X-ray diffraction, imaging, editorial

## Abstract

This issue of *Journal of Applied Crystallography* includes some highlights of the 11th Biennial Conference on High-Resolution X-ray Diffraction and Imaging (XTOP), held in St Petersburg in 2012.

This special issue of *Journal of Applied Crystallography* includes some highlights of the 2012 Conference on High-Resolution X-ray Diffraction and Imaging (XTOP). The 11th Biennial Conference was held in St Petersburg, Russia, by the Ioffe Physical Technical Institute of the Russian Academy of Sciences, the Russian National Committee for Crystallography, the A. V. Shubnikov Institute of Crystallography and the National Research Centre ‘Kurchatov Institute’. This successful conference series started in Marseille (1992), with subsequent meetings in Berlin, Palermo, Durham, Ustron-Jaszowiec, Grenoble, Prague, Karlsruhe, Linz and most recently Warwick in 2010.

The original goal of this conference series was to discuss the development of modern techniques in X-ray diffraction analysis and the application of these methods to study structural properties of crystalline objects. The conference subject matter has been steadily expanding to include X-ray standing waves, refraction tomography, X-ray reflectometry *etc.*, in addition to the conventional methods of X-ray diffraction analysis and topography. The development of research techniques has been facilitated by the increasingly wide use of synchrotron radiation and computer processing. As a consequence, topics devoted to X-ray optics, coherent imaging, time-resolved diffraction *etc*. have appeared in the programmes of the XTOP symposia.

The range of the objects under study has expanded and now includes single crystals, surface crystalline layers, nanostructures and biomaterials. The technological development of new materials and structures, including nano-size systems and particles, has required a detailed and exhaustive knowledge of the structural state of these objects, which has stimulated further intensive development of X-ray research techniques. It is the solution to these challenges that was the essence of XTOP 2012. The highlights presented here include a good cross section of the activities in this field, from ordering in polymers, through *in situ* measurements on colloidal crystals, to epitaxial layer characterization (including relaxation and quantum dot analysis), nanoparticles and nanowires, and studies on defects and crack propagation. There are also articles on the theory of diffraction from distorted crystals, methods in micro-tomography and its use in biological studies, and new ideas in crystal optics to condition X-rays to improve data for the future.

Over 220 participants from more than 20 countries attended this conference, which lasted for four-and-a-half days and included six tutorial lectures, nine invited talks, about 60 oral presentations and three poster sessions with about 150 posters. The highlights included in this special issue give a representative view of the conference series and those four-and-a-half days in St Petersburg in September 2012.

